# Connection between Oligomeric State and Gating Characteristics of Mechanosensitive Ion Channels

**DOI:** 10.1371/journal.pcbi.1003055

**Published:** 2013-05-16

**Authors:** Christoph A. Haselwandter, Rob Phillips

**Affiliations:** 1 Department of Physics and Astronomy, University of Southern California, Los Angeles, California, United States of America; 2 Department of Applied Physics, California Institute of Technology, Pasadena, California, United States of America; Rutgers University, United States of America

## Abstract

The mechanosensitive channel of large conductance (MscL) is capable of transducing mechanical stimuli such as membrane tension into an electrochemical response. MscL provides a widely-studied model system for mechanotransduction and, more generally, for how bilayer mechanical properties regulate protein conformational changes. Much effort has been expended on the detailed experimental characterization of the molecular structure and biological function of MscL. However, despite its central significance, even basic issues such as the physiologically relevant oligomeric states and molecular structures of MscL remain a matter of debate. In particular, tetrameric, pentameric, and hexameric oligomeric states of MscL have been proposed, together with a range of detailed molecular structures of MscL in the closed and open channel states. Previous theoretical work has shown that the basic phenomenology of MscL gating can be understood using an elastic model describing the energetic cost of the thickness deformations induced by MscL in the surrounding lipid bilayer. Here, we generalize this elastic model to account for the proposed oligomeric states and hydrophobic shapes of MscL. We find that the oligomeric state and hydrophobic shape of MscL are reflected in the energetic cost of lipid bilayer deformations. We make quantitative predictions pertaining to the gating characteristics associated with various structural models of MscL and, in particular, show that different oligomeric states and hydrophobic shapes of MscL yield distinct membrane contributions to the gating energy and gating tension. Thus, the functional properties of MscL provide a signature of the oligomeric state and hydrophobic shape of MscL. Our results suggest that, in addition to the hydrophobic mismatch between membrane proteins and the surrounding lipid bilayer, the symmetry and shape of the hydrophobic surfaces of membrane proteins play an important role in the regulation of protein function by bilayer membranes.

## Author Summary

A fundamental property of living cells is their ability to detect mechanical stimuli. Microbes, in particular, often transition between different chemical environments, leading to osmotic shock and concurrent changes in membrane tension. The tension of microbial cell membranes is detected and controlled by membrane molecules such as the widely-studied mechanosensitive channels which, depending on the tension exerted by the surrounding lipid bilayer, switch between closed and open states. Thus, the biological function of mechanosensitive channels relies on an interplay between bilayer mechanical properties and protein structure. Using a physical model of cell membranes it was shown previously that the basic phenomenology of mechanosensitive gating can be understood in terms of the bilayer deformations induced by mechanosensitive channels. We have generalized this physical model to allow for the molecular structures of mechanosensitive channels reported in recent experiments. Our methodology allows the calculation of protein-induced membrane deformations for arbitrary oligomeric states of membrane proteins. We predict that distinct oligomeric states and hydrophobic shapes of mechanosensitive channels lead to distinct functional responses to membrane tension. Our results suggest that the shape of membrane proteins, and resulting structure of membrane deformations, plays a crucial role in the regulation of protein function by bilayer membranes.

## Introduction

The biological function of membrane proteins is determined by a complex interplay between protein structure and the properties of the surrounding lipid bilayer [1]–[6]. In particular, the bilayer hydrophobic core couples to the hydrophobic regions of membrane proteins [7]–[10]. The resulting deformations in the lipid bilayer membrane from its unperturbed state can be described quantitatively [11]–[17] using the continuum elasticity theory of membranes [18]–[20]. The energetic cost of protein-induced membrane deformations depends on the protein conformational state as well as on the bilayer material properties, which allows [11]–[17] the lipid bilayer to act as a regulator of protein function. A widely-studied model system for the coupling between membrane protein function and the elastic deformation of lipid bilayers is provided by mechanosensitive ion channels. Mechanosensitive channels are capable of transducing membrane tension into an electrochemical response [21]–[23] by switching from a closed to an open conformational state with increasing membrane tension, allowing cells to sense touch, sound, and pressure.

A paradigm of mechanosensation is the prokaryotic mechanosensitive channel of large conductance (MscL) [24]–[26]. In particular, biophysical approaches such as patch-clamp experiments and reconstitution of MscL in artificial lipid bilayer vesicles have allowed [24]–[34] a systematic analysis of the relation between lipid material properties and the gating probability of MscL with increasing membrane tension. However, despite its central significance, even basic issues such as the physiologically relevant oligomeric states and molecular structures of MscL remain a matter of debate [26], [35]–[37]. So far, the oligomeric state and molecular structure of MscL have mainly been studied [24]–[27], [33], [35]–[47] using crystallographic, biochemical, and computational approaches. This has led to the identification of a number of possible oligomeric states and molecular structures of MscL. In particular, early low-resolution electron microscopy studies suggested that MscL is a hexamer [39], while more recent high-resolution x-ray crystallographic studies demonstrated pentameric [40] and tetrameric [46] MscL structures. Do the various reported stoichiometries of MscL induce distinct membrane deformations, yielding distinct functional responses to membrane tension? More generally, theoretical studies of the energetic cost of protein-induced membrane deformations [11]–[15] have mostly focused on membrane inclusions with a cylindrical or conical hydrophobic shape. But experimental surveys of the protein content in the membranes of, for instance, synaptic vesicles [48] and *Acinetobacter baumannii* [49] suggest [50] that membrane proteins exhibit great diversity in their oligomeric state and transmembrane shape. What is the relationship between the oligomeric state and hydrophobic shape of a membrane protein and the elastic energy required to accommodate the membrane protein within the lipid bilayer?

In this article we address the above questions on the basis of the continuum elasticity theory of lipid bilayer membranes [18]–[20]. In particular, we generalize the standard framework for calculating the energetic cost of protein-induced membrane deformations [11]–[15], which was employed previously to understand the basic phenomenology of MscL gating [51]–[54], to account for non-circular cross sections of membrane proteins. Our methodology establishes a quantitative relationship between the oligomeric state and hydrophobic shape of a membrane protein and the elastic energy required to accommodate the membrane protein within the lipid bilayer membrane. We make quantitative predictions pertaining to the gating characteristics associated with various structural models of MscL and, in particular, show that different oligomeric states and hydrophobic shapes of MscL yield distinct membrane contributions to the gating energy and gating tension. Generally we find that the oligomeric state and hydrophobic shape of a membrane protein are reflected in the energetic cost of the lipid bilayer deformations necessary to accommodate the protein within the membrane. Our results suggest that, in addition to the hydrophobic mismatch between membrane proteins and the surrounding lipid bilayer [11]–[15], the symmetry and shape of the hydrophobic surfaces of membrane proteins play an important role in the regulation of protein function by bilayer membranes. The results and predictions of our model calculations are described in the Results and Discussion sections. The Models and Methods section provides a detailed mathematical formulation of our analytic methodology linking the hydrophobic shape of membrane proteins to the elastic deformations in the surrounding lipid bilayer membrane.

## Results

### Phenomenology of mechanosensitive gating

The basic experimental phenomenology of mechanosensitive gating is captured by a two-state Boltzmann model [27]–[33] describing the competition between the closed and open states of MscL. The central quantity in this model is the channel opening probability
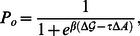
(1)where 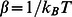, in which 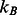 is Boltzmann's constant and 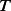 is the temperature, 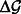 is the total free energy difference between the open and closed states of MscL, 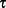 is the membrane tension, and 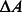 is the area difference between the open and closed channel states. Equation (1) implies that, for a fixed 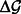, a given channel is more likely to be in the open state for larger values of the membrane tension and provides a simple description of experimental data on MscL gating [14], [25], [27]–[33], although a more detailed description of MscL gating would need to take into account the existence of multiple conductance states [31], [33], [43], [44].

A deeper understanding of Eq. (1) in terms of the physical mechanisms underlying MscL gating hinges on a quantitative description of the various contributions to 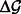. To this end it is useful [51]–[54] to write  as the sum of protein and lipid bilayer contributions,
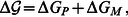
(2)where 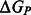 denotes the difference in internal protein free energy between the open and closed channel states, and 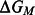 denotes the difference in membrane deformation energy between the open and closed states. In general, 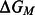 depends on the oligomeric state and hydrophobic shape of MscL in the closed and open channel states, as well as on bilayer material properties such as the bilayer hydrophobic thickness and bending rigidity. In the remainder of this article we focus on the membrane deformations induced by MscL. To simplify our notation we therefore drop the subscript 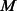 in 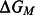 and denote by 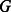 the membrane deformation energy associated with MscL.

The continuum elasticity theory of membranes [18]–[20] provides a general framework for evaluating bilayer-protein interactions [11]–[15], [55]–[63] and, hence, the membrane contribution in Eq. (2). On this basis, the elastic membrane deformations required to accommodate MscL within the bilayer membrane were estimated previously [51]–[54] under the assumption that the transmembrane region of MscL is cylindrical in the closed and open channel states. In particular, it was found that thickness deformations 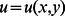, where 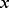 and 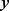 are spatial coordinates along the bilayer membrane, are the dominant elastic membrane deformations induced by MscL. The quantitative details of this previous model of MscL gating, which forms the foundation for the work presented here, are summarized in the Models and Methods section. The overall conclusion of the cylinder model of MscL [51]–[54] is that 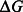 can be of the same order of magnitude as the measured values of 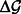 [27], [29], [30], [33] in Eq. (1), with both 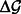 and 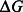 being (much) larger than the thermal energy. This suggests that membrane mechanics plays a central role in mechanotransduction and the biological function of MscL. This conclusion is also consistent with experiments measuring the dependence of MscL gating on membrane composition [27], [34]. We emphasize, however, that in general the protein contribution to the free energy difference in Eq. (2) must be considered, and may very well dominate over the membrane contribution. The calculation of the membrane contribution to the gating energy merely represents one step in drawing up a general energy budget of gating.

As mentioned above, the determination of the oligomeric state and, more generally, molecular structure of MscL in different conformational states is a problem of intense experimental interest [24]–[27], [33], [35]–[47]. How do the observed discrepancies in the oligomeric state and molecular structure of MscL relate to the mechanosensitive gating characteristics relevant for the biological function of MscL? In order to address this question from the perspective of membrane mechanics we formally divide 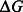 into two contributions,
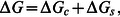
(3)where 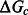 corresponds to the membrane deformation energy associated with the idealized cylinder model of MscL [14], [51]–[54], which we employ as our point of reference when estimating the membrane deformations induced by different oligomeric states of MscL, and 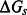 corresponds to the modification of 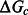 due to deviations of the hydrophobic cross section of MscL from the circle. In particular, 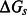 depends on the oligomeric state (symmetry) of MscL. We have obtained the analytic solution of the general elastic equations describing bilayer deformations induced by MscL in the limit of weak perturbations about the cylindrical reference shape, thus providing a general framework for estimating 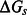 for arbitrary oligomeric states. The mathematical details of these calculations are described in the Models and Methods section. As discussed below, we find that the oligomeric state and hydrophobic shape of MscL can have a considerable effect on the membrane deformation energy. Thus, based on the membrane deformation energy, distinct boundary shapes and, in particular, distinct oligomeric states of MscL are predicted to yield distinct mechanosensitive gating curves.

### Hydrophobic shape of mechanosensitive channels

**Figure 1:**
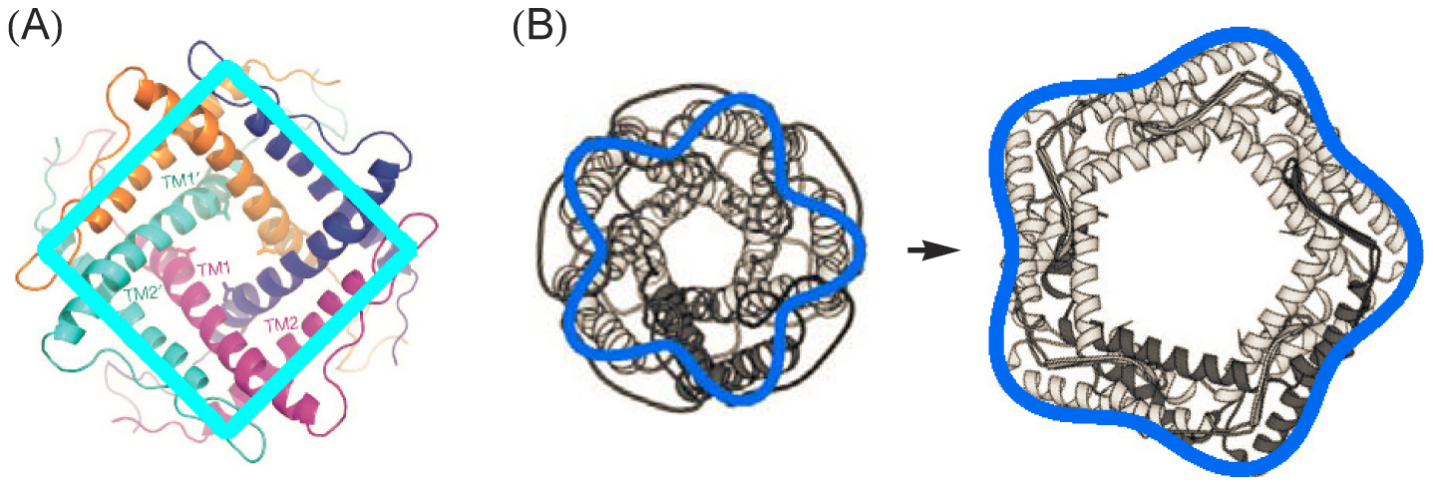
Molecular models and boundary curves of the cross section of MscL viewed along the pore axis. (A) Tetrameric structure of SaMscL obtained via x-ray crystallography [46]. (B) Pentameric structure of the closed state of MtMscL obtained via x-ray crystallography [40] (left panel) and pentameric open-state structure of MtMscL proposed in Refs. [43], [44] (right panel). For a further discussion see Ref. [45]. The contour lines superimposed on the molecular models denote the corresponding shapes of the boundary curve 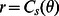 used in our elastic model of bilayer deformations. See the Models and Methods section for further details. Molecular models reprinted, with permission, from Nature Publishing Group [46] (Panel A) and The Annual Review of Biophysics and Biomolecular Structure [45] (Panel B).

A variety of different approaches have been employed [24]–[27], [33], [35]–[47] to study the molecular structure of MscL in different conformational states. Figure 1 shows examples of the molecular structures of MscL obtained for *Staphylococcus aureus* (SaMscL) and *Myobacterium tubercolosis* (MtMscL). In particular, Fig. 1(A) displays the tetrameric structure of SaMscL solved most recently [46] using x-ray crystallography. This structure may correspond to an expanded state which is intermediate between the closed and open states of MscL. Figure 1(B) shows pentameric structures of the closed and open states of MscL proposed for MtMscL using crystallographic, biochemical, and computational approaches. The closed state of MscL displayed in the left-hand panel of Fig. 1(B) was obtained on the basis of x-ray crystallography [40], while the right-hand panel displays a molecular model suggested for the open state of MscL [43]–[45]. For MscL in *Escherichia coli* (EcoMscL), hexameric [38], [39] as well as pentameric [43]–[45] molecular models have been proposed.

The contour lines approximating the cross sections of the transmembrane domains in Fig. 1 represent the bilayer-MscL boundary curves 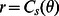 used in our membrane-mechanical model of MscL gating. Similar fits are obtained for the hexameric [38], [39] and pentameric [43]–[45] models proposed for EcoMscL (in particular, see Fig. 3 in Ref. [39] and Fig. 5 in Ref. [44]). The subscript 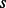 in 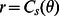 denotes the oligomeric state (symmetry) of MscL with tetrameric, pentameric, and hexameric structures of MscL corresponding to 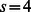, 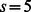, and 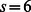, respectively. As discussed further in the Models and Methods section, we express the bilayer-MscL boundary curves in terms of the variables 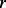 and 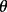, which are the radial coordinate and the polar angle associated with a polar coordinate system having the MscL protein at its center. The cylinder model of MscL [51]–[54] corresponds to choosing 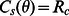 and 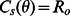 in the closed and open states of MscL, where 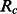 and 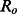 are the cylinder radii in the closed and open channel states. However, as apparent from Fig. 1, the proposed hydrophobic cross sections of MscL [24]–[27], [33], [35]–[47] often deviate from a circle. Indeed, inspired by the structural models of MscL in Fig. 1 and Refs. [38], [39], [43]–[45], we distinguish between two basic shapes of boundary curves. The “polygonal boundary curves” correspond to the tetragonal boundary curve shown in Fig. 1(A) (see Fig. 5 in Ref. [44] for examples of pentagonal boundary curves), while the “clover-leaf boundary curves” correspond to the pentameric propeller shapes in Fig. 1(B) (see Fig. 3 in Ref. [39] for examples of hexameric clover-leaf shapes).

Following the approach summarized in Eq. (3), we employ the cylinder model of MscL [51]–[54] as a means to isolate the role played by the oligomeric state and hydrophobic shape of MscL [24]–[27], [33], [35]–[47] in the regulation of MscL by the surrounding lipid bilayer. In particular, in the simplest model of MscL the hydrophobic thickness of MscL is assumed to be constant when transitioning between closed and open channel states [51], [52], while a more general model [53], [54] allows for changes in the hydrophobic thickness of MscL [43], [44], [47]. We consider here both models of the hydrophobic thickness of MscL but, to systematically study the role played by MscL shape in MscL gating, focus on the case of a constant hydrophobic thickness (see the Models and Methods section for details). In either case we always use the same hydrophobic thickness when making comparisons between different shapes of MscL so as to isolate the role played by MscL shape. Moreover, in order to compare membrane inclusions of equal size, and in light of the central role played by the protein area in Eq. (1), we generally contrast different oligomeric states and hydrophobic shapes of MscL for a fixed area of the hydrophobic cross section. This assumption allows us to make direct comparisons with previous work on bilayer-MscL interactions [51]–[54], and eliminates any spurious effects resulting from MscL occupying different membrane areas in different oligomeric states, but would need to be relaxed for a more detailed description of the membrane deformations induced by MscL. In particular, we use for the closed and open states of MscL the cross-sectional areas 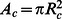 and 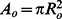 with 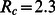 nm and 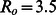 nm, which were estimated previously [51], [52], [54] for the cylinder model of MscL on the basis of the available structural models of MscL [27], [33], [40]–[45], [47]. Setting the cross-sectional area equal to 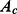 or 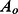 fixes the size of the polygonal and clover-leaf shapes, with all other parameters in 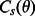 determined by the respective symmetries and morphologies of the MscL boundary curves. For comparison, we also consider polygonal shapes having the same circumference, rather than the same area, as the cylindrical reference shape in the closed and open channel states.

### Structure of elastic membrane deformations

**Figure 2:**
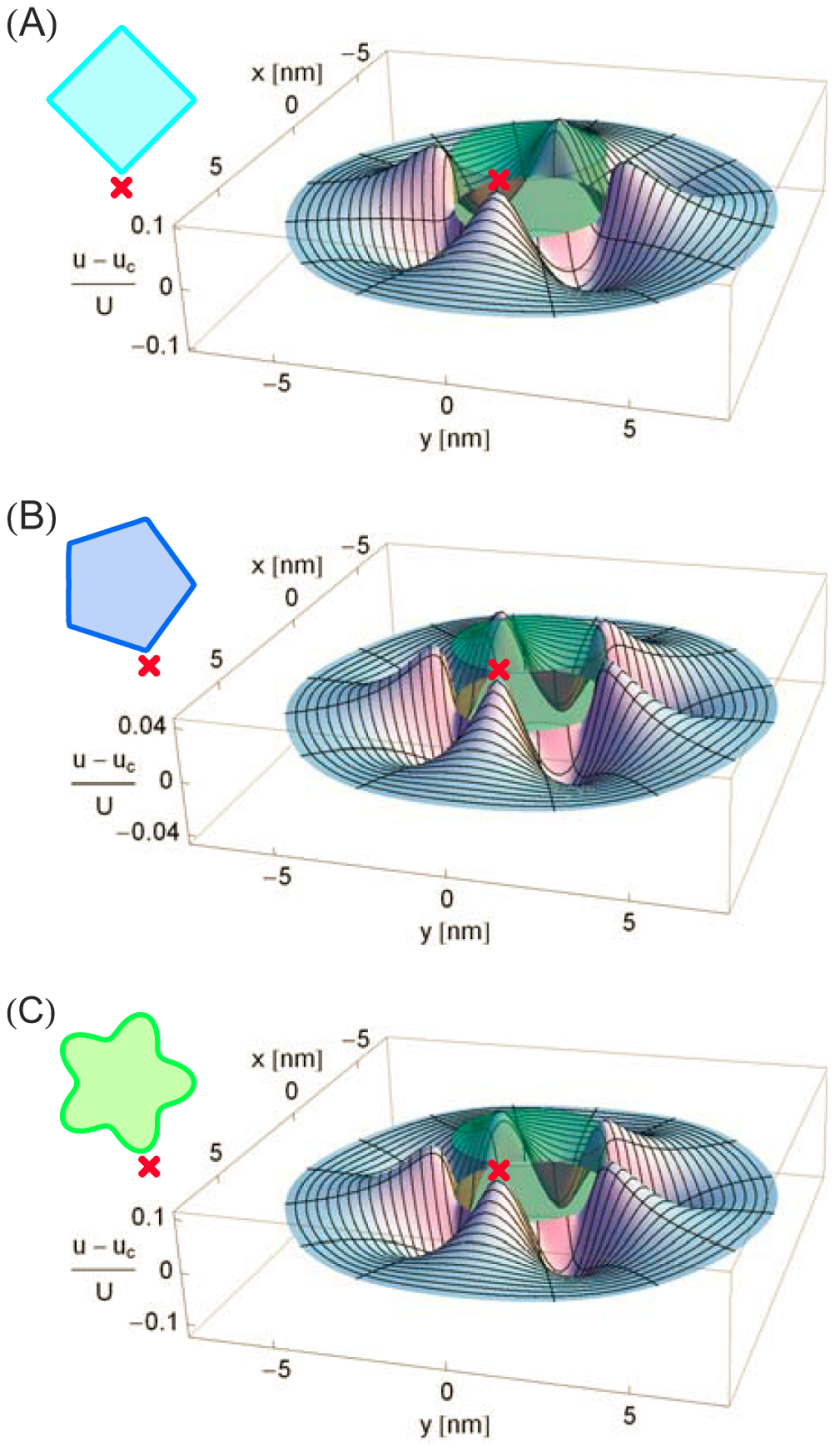
Membrane deformations induced by selected structural models of MscL. Difference in thickness deformation profile, 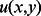, due to (A) the tetragonal structure of MscL in Fig. 1(A), (B) the pentagonal structure of MscL proposed in Ref. [44], and (C) the pentameric clover-leaf structure of MscL in the left-hand panel of Fig. 1(B) and the thickness deformation profile due to a cylindrical membrane inclusion (indicated by a partially transparent cylinder), 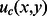, normalized by the hydrophobic mismatch between MscL and the bilayer membrane, 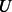. All membrane inclusions have a cross-sectional area 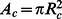 and a hydrophobic thickness corresponding to the closed state of MscL [51], [52]. To calculate differences in membrane deformation fields we mapped the boundary conditions associated with non-cylindrical inclusion shapes onto equivalent boundary conditions for cylindrical inclusions of variable hydrophobic thickness [see Eqs. (27) and (28) in the Models and Methods section for quantitative details]. The relative orientations of the MscL shapes shown in the insets and the corresponding bilayer deformations are indicated by crosses.

Figure 2 shows the difference in the membrane deformation fields induced by some of the structural models of MscL in Fig. 1 and Refs. [39], [44] and the cylinder model of MscL [51]–[54]. As described in greater detail in the Models and Methods section, we estimated the membrane deformation field due to a given oligomeric state and molecular structure of MscL by minimizing the elastic membrane energy with respect to the thickness deformation field 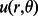 in the limit of weak deviations from the cylindrical reference shape. In particular, Figs. 2(A), 2(B), and 2(C) show the difference in the thickness deformation fields induced by the tetragonal, pentagonal, and pentameric clover-leaf models of MscL in Fig. 1 and Ref. [44] and the cylinder model of MscL. The cross sections of all membrane inclusions in Fig. 2 are of the area 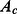 corresponding to the closed state of the cylinder model of MscL. The deformation profiles in Fig. 2 demonstrate that the symmetry and shape of the hydrophobic surface of a membrane protein are reflected in the structure of the membrane deformations required to accommodate the protein within the lipid bilayer.

Figure 2 allows us to gain some intuition regarding the membrane deformations associated with different oligomeric states and hydrophobic shapes of MscL. First consider the deformation fields in Figs. 2(A) and 2(B) due to polygonal boundary curves. Tetragonal and pentagonal boundary curves yield membrane deformations exhibiting four- and five-fold symmetry, respectively. However, while polygonal boundary curves of four-fold and lower-order symmetry produce considerable deviations from the deformation field of the cylindrical reference shape, the shallow angles of pentagonal boundary curves only produce relatively small deviations. Indeed, for hexagonal and higher-order symmetries the deviations from the cylindrical deformation field are even smaller than those shown in Fig. 2(B). For clover-leaf shapes, however, the overall deviation from the deformation field induced by the cylinder model of MscL increases with increasing symmetry of the oligomeric state. As illustrated in Fig. 2(C), clover-leaf shapes of pentameric and higher-order symmetry can, in addition to clover-leaf shapes of lower-order symmetry, yield substantial modifications of the deformation field associated with cylindrical membrane inclusions. Thus, for the polygonal structures of MscL in Fig. 1 and Refs. [39], [44] the overall deviation from the elastic deformation footprint of the cylinder model of MscL decreases with increasing symmetry, but for clover-leaf shapes the overall deviation becomes more pronounced with increasing symmetry.

### Membrane deformation energy: Mechanosensitive channels

**Figure 3:**
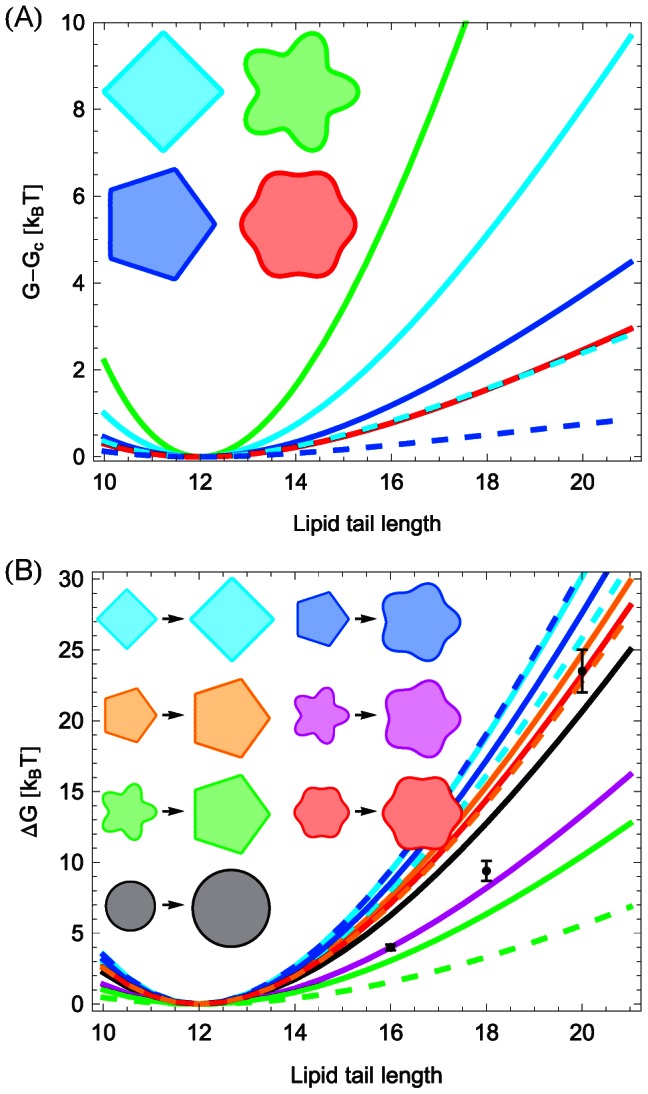
Membrane deformation energy induced by selected structural models of MscL. (A) Difference in thickness deformation energy associated with the structural models of the closed state of MscL in Fig. 1 and Refs. [39], [44], 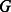, and the cylinder model of MscL, 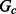, as a function of PC lipid tail length. (B) Difference in thickness deformation energy between the open and closed states of MscL as a function of PC lipid tail length for the structural models of the closed state of MscL in Fig. 1 and Refs. [39], [44], for a closed pentagonal shape and an open pentameric clover-leaf shape of MscL, for a closed pentameric clover-leaf shape and an open pentagonal shape of MscL, and for the cylinder model of MscL. The filled circles with error bars denote the total free energy differences between the open and closed channel states, 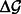, estimated by Perozo *et al.* [27] for EcoMscL. The solid curves in panels (A) and (B) correspond to membrane inclusions with cross-sectional area 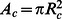 or 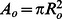, respectively, while the dashed curves correspond to polygonal shapes with circumference 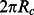 or 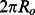. We used identical values of the hydrophobic thickness of MscL for all channel shapes and states [51], [52], and related lipid tail length to bilayer hydrophobic thickness as described in Ref. [51]. See the Models and Methods section for further quantitative details.

Figure 3(A) shows the difference in membrane deformation energy between some of the structural models of MscL in Fig. 1 and Refs. [39], [44] and the cylinder model of MscL [51]–[54] as a function of lipid tail length (bilayer hydrophobic thickness). Irrespective of the oligomeric state or hydrophobic shape of MscL, deviations of the cross section of MscL from the circle, and the corresponding non-trivial structure of the membrane deformation field, are seen to increase the elastic energy required to embed MscL within the bilayer membrane. Consistent with the deformation profiles in Fig. 2, the elastic energy difference between polygonal shapes of MscL and the cylinder model of MscL is largest for the tetragonal structure in Fig. 1(A) and decreases with increasing symmetry of the oligomeric state, with hexagonal and higher-order boundary curves inducing elastic membrane deformations of essentially the same energetic cost as the cylinder model of MscL. These conclusions do not change if we consider polygonal models of MscL which have the same circumference, rather than the same cross-sectional area, as the cylindrical reference shape. The pentameric clover-leaf shape of MscL in the closed state [see Fig. 1(B)] induces membrane deformations which carry a greater energetic cost than any of the polygonal shapes considered in Fig. 3(A). In contrast, due to its decreased deviation from the cylindrical reference shape, the hexameric clover-leaf shape of MscL in Ref. [39] carries a relatively small cost in membrane deformation energy. Overall, Fig. 3(A) shows that the various structural models of MscL proposed in previous studies [24]–[27], [33], [35]–[47], and the polygonal or clover-leaf boundary shapes associated with these structural models, yield considerable differences in the membrane deformation energy required to embed MscL within a lipid bilayer membrane.

In Fig. 3(B) we compare the elastic energy difference between the open and closed states of MscL for the structural models of MscL gating in Fig. 1 and Refs. [39], [44] (tetragonal shapes in light blue, pentagonal shapes in orange, pentameric clover-leaf shapes in purple, and hexameric clover-leaf shapes in red) and the cylinder model of MscL [51]–[54] (black). For completeness, we also consider in Fig. 3(B) transitions between a closed pentagonal shape and an open pentameric clover-leaf shape of MscL (dark blue), as well as the reverse case of transitions between a closed pentameric clover-leaf shape and an open pentagonal shape of MscL (green). For all of these plots we used the parameter values characterizing bilayer-MscL interactions estimated in Refs. [51], [52] with zero membrane tension. As discussed in greater detail in the Models and Methods section, this parameterization of bilayer-MscL interactions allows the systematic study of the effect of the structure of membrane deformations on the gating characteristics of MscL, without the further complications introduced by MscL having different hydrophobic thicknesses in the closed and open channel states. We also include in this plot the total free energy differences between the open and closed states of EcoMscL estimated by Perozo *et al.* [27] for PC16, PC18, and PC20 bilayers at zero membrane tension. In the case of transitions between the polygonal structures in Fig. 1 and Ref. [44], we again find that the deviation from the cylindrical reference shape is more pronounced for tetragonal shapes than for pentagonal shapes, and that in either case the free energy of gating is increased relative to cylindrical membrane inclusions.

In addition, Fig. 3(B) shows that, for transitions between the pentameric clover-leaf shapes in Fig. 1, the difference in membrane deformation energy between the open and closed states of MscL is strongly decreased relative to cylindrical inclusions. We attribute this to the larger deformation of the circular boundary curve for the closed pentameric clover-leaf shape in Fig. 1(B) [see also Fig. 3(A)] as compared to the corresponding open pentameric clover-leaf shape. Allowing for (hypothetical) transitions between different families of boundary curves, the situation becomes more complex. Transitions from a closed pentagonal to an open pentameric clover-leaf shape show a strongly increased gating energy, whereas transitions from a closed pentameric clover-leaf shape to an open pentagonal shape carry a small penalty as far as the elastic membrane deformation energy is concerned. This trend is amplified if pentagonal shapes of the same circumference, rather than of the same cross-sectional area, as the cylindrical reference shape are considered. In summary, Fig. 3(B) indicates that, for the proposed structural models of MscL gating [24]–[27], [33], [35]–[47], the term 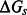 in Eq. (3) is generally of the same order of magnitude as 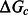, with different structural models of MscL displaying a characteristic dependence of the sign and numerical value of 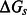 on the bilayer hydrophobic thickness.

### Membrane deformation energy: Systematic trends

**Figure 4:**
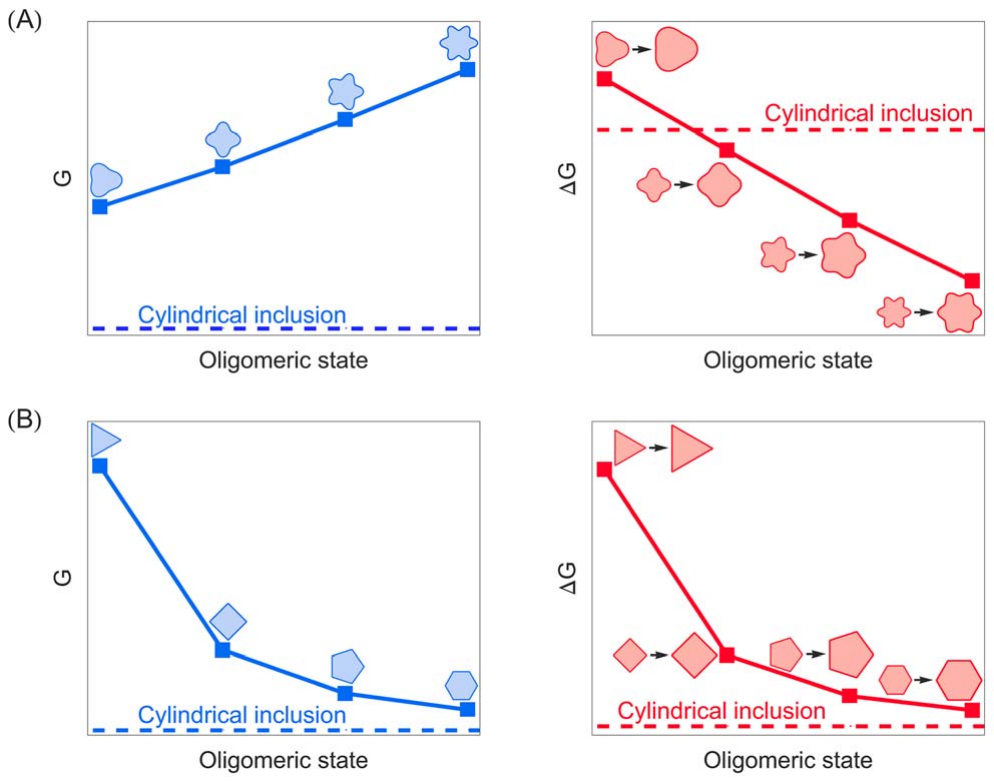
Variation of membrane deformation energy and gating energy with protein oligomeric state. Schematic illustration of the dependence of the thickness deformation energy 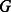 (left column) and gating energy 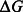 (right column) on the protein oligomeric state for (A) clover-leaf shapes and (B) polygonal shapes. We considered variations in oligomeric state from trimers to hexamers, and used boundary shapes inspired by the structural models of MscL in Fig. 1 and Refs. [39], [44]. For each data point, the corresponding inclusion shape (left panels) or sequence of inclusion shapes (right panels) is illustrated schematically. For comparison we also show the membrane deformation energy and gating energy associated with the cylinder model of MscL [51]–[54]. The cross-sectional areas of the inclusions in the left-hand panels correspond to the closed state of MscL, while the two inclusion sizes at each data point in the right-hand panels correspond to the open and closed states of MscL. We used identical values of the hydrophobic inclusion thickness for all shapes and states shown. See Figs. S1, S2, S3 and the Models and Methods section for mathematical details.

Figure 4 provides a systematic comparison of the membrane deformation energy associated with different oligomeric states of MscL for the polygonal and clover-leaf boundary shapes inspired by the molecular models in Fig. 1 and Refs. [39], [44] (see Fig. S1). As in Fig. 3B, we used for Fig. 4 the same hydrophobic mismatch for closed and open states of MscL [51], [52]. For the clover-leaf shapes in Fig. 4 we considered shapes which were perturbed by the same amplitude about the cylindrical reference shape in open and closed states. The left-hand panel of Fig. 4(A) shows a clear progression in membrane deformation energy as a function of the oligomeric protein state, with lower-order clover-leaf shapes being energetically favorable compared to higher-order clover-leaf shapes. All clover-leaf shapes induce a membrane deformation energy which is greater than the deformation energy associated with the cylinder model of MscL [see Fig. S2(A) for more comprehensive results]. The elastic energy differences between the open and closed states of clover-leaf shapes are displayed in the right-hand panel of Fig. 4(A). We find that the gating energy of clover-leaf shapes decreases with increasing channel symmetry. Intriguingly, oligomeric states of high enough symmetry yield a gating energy which is reduced relative to cylindrical inclusions of the same cross-sectional area (see Fig. S3 for more comprehensive results).

The left-hand panel of Fig. 4(B) illustrates the membrane deformation energy of the closed state of MscL for trigonal, tetragonal, pentagonal, and hexagonal boundary curves. In contrast to clover-leaf shapes, the membrane deformation energy corresponding to polygonal inclusion shapes decreases with increasing symmetry, and eventually approaches the deformation energy associated with cylindrical inclusions. For membrane inclusions of equal circumference the convergence of the membrane deformation energies induced by polygonal and cylindrical inclusions is rendered more rapid as compared to membrane inclusions of the same cross-sectional area [see Fig.S2 (B)]. The elastic energy differences between the open and closed states of polygonal boundary curves are illustrated in the right-hand panel of Fig. 4(B), and exhibit characteristics which are qualitatively different from the corresponding results for clover-leaf shapes in the right-hand panel of Fig. 4(A). For polygonal shapes the energy difference between the open and closed channel states decreases with increasing symmetry of the membrane inclusion, and is always greater than the elastic gating energy associated with the cylindrical reference shape. These conclusions hold for membrane inclusions of equal circumference as well as inclusions of the same cross-sectional area (see Fig. S3). Polygonal boundary curves with six-fold or higher-order symmetry yield, for the parameter values appropriate for MscL [51], [52], a gating energy which closely approaches the corresponding gating energy associated with the cylinder model of MscL (see Fig. S3 for more comprehensive results). Thus, Fig. 4 predicts systematic trends in the total membrane deformation energy required to accommodate MscL (or other membrane proteins with comparable hydrophobic surfaces) within the bilayer membrane, and in the elastic gating energy, as the oligomeric state and protein shape are being varied.

### Gating curves: Mechanosensitive channels

We now turn to the dependence of the channel opening probability in Eq. (1) on the oligomeric state and hydrophobic shape of MscL. It should be emphasized that we thereby focus solely [11]–[15], [51]–[54] on the lipid bilayer contribution to the total free energy difference between the open and closed channel states, and neglect any contributions to the gating energy due to changes in the internal protein conformation. While it was argued previously [51]–[54] that, in certain situations, the total free energy difference between the open and closed states of MscL can be of the same order of magnitude as the difference in membrane deformation energy between the open and closed states of MscL, other contributions to the free energy difference must generally be considered. Note, however, that our results in Fig. 3(B) indicate that the term 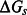 in Eq. (3) capturing contributions to the membrane deformation energy due to deviations of the hydrophobic cross section of MscL from the circle is generally of the same order of magnitude as the elastic energy difference 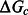 calculated previously using the cylinder model of MscL [51]–[54]. Thus, the structure of lipid bilayer deformations associated with different oligomeric states and shapes of MscL is expected to affect the gating characteristics of MscL.

**Figure 5:**
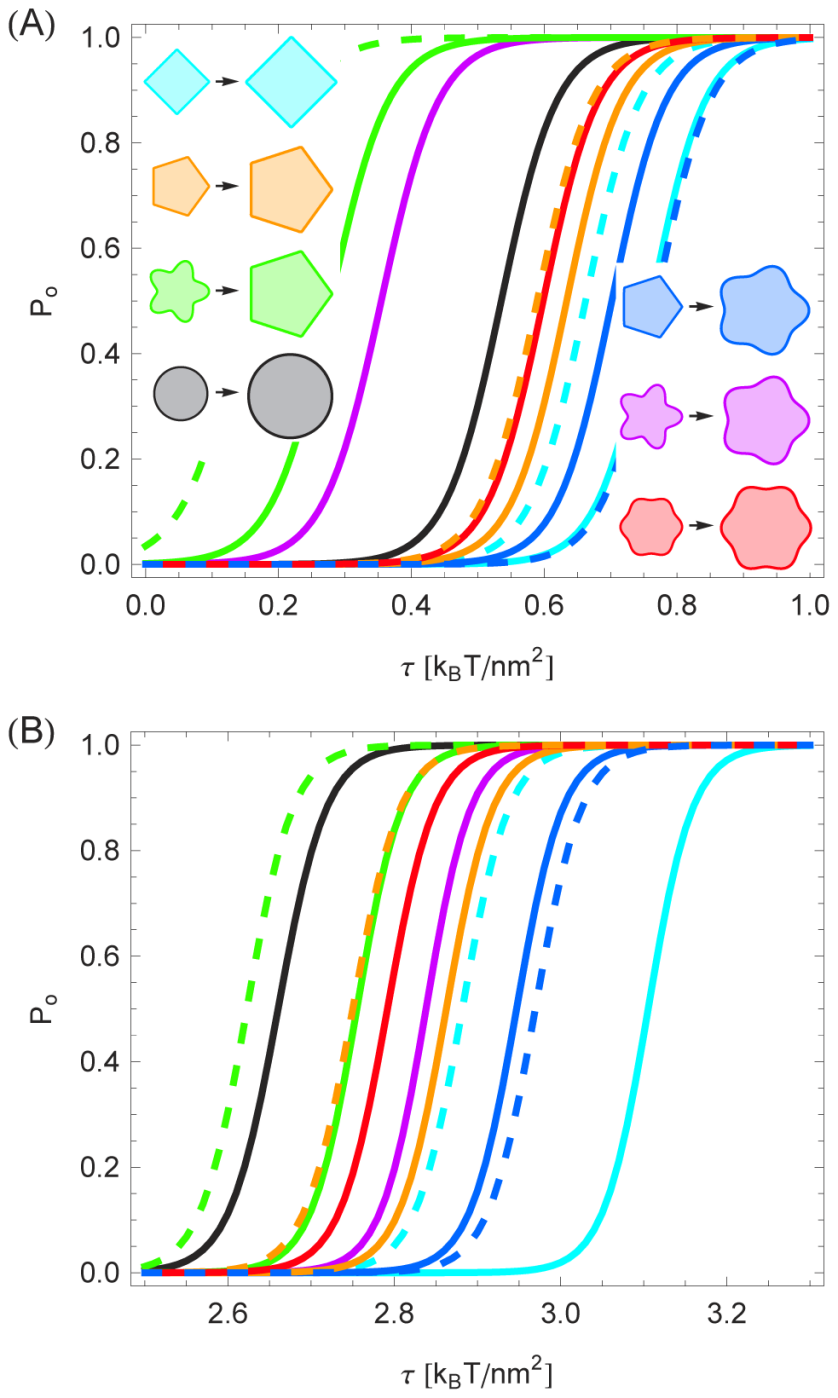
Membrane contribution to the gating probability of selected structural models of MscL. Opening probability of MscL in Eq. (1) for the structural models of MscL in Fig. 1 and Refs. [39], [44], for a closed pentagonal shape and an open pentameric clover-leaf shape of MscL, for a closed pentameric clover-leaf shape and an open pentagonal shape of MscL, and for the cylinder model of MscL calculated using (A) identical values of the hydrophobic thickness of MscL in the closed and open channel states [51], [52] and (B) the distinct values of the hydrophobic thickness of MscL in the closed and open channel states suggested [53], [54] by structural studies of MscL [40], [41], [47]. The solid curves denote membrane inclusions with cross-sectional area 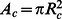 or 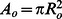, respectively, while the dashed curves denote polygonal shapes with circumference 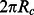 or 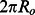. See the Models and Methods section for further quantitative details.

In order to facilitate the systematic investigation of the connection between the oligomeric state and the gating energy of MscL in Fig. 3(B) we employed the parameterization of bilayer-MscL interactions in Refs. [51], [52] and used the same hydrophobic mismatch for closed and open states of MscL. Applying these parameter values to the fits to the structural models in Fig. 1 and Refs. [39], [44] we found the gating curves shown in Fig. 5(A). The tetragonal model of MscL in Fig. 1(A) is seen to gate at a larger tension than the pentagonal model of MscL in Ref. [44], with both models yielding a larger gating tension than the cylindrical reference shape. In contrast, the pentameric clover-leaf model of MscL in Fig. 1(B) produces a smaller gating tension than the hexameric clover-leaf model of MscL, the cylinder model of MscL, as well as the tetragonal and pentagonal models of MscL. Moreover, for a pentagonal shape of MscL in the closed state and a pentameric clover-leaf shape in the open state, Fig. 5(A) predicts a relatively large gating tension. In contrast, the reverse case of a pentameric clover-leaf shape in the closed state and a pentagonal open state yields a markedly smaller gating tension than any of our other models of MscL gating motivated by Fig. 1 and Refs. [39], [44].

Figure 5(B) displays the same gating curves as Fig. 5(A), but using the distinct values of the hydrophobic thickness of the closed and open states of MscL suggested by structural studies of MscL [40], [41], [47]. In this parameterization of bilayer-MscL interactions [53], [54], closed and open states of MscL are distinguished not only by their hydrophobic cross section but also by their hydrophobic thickness. As a result, gating is driven by a more complex interplay between the energetics of thickness deformations and the structure of membrane deformations induced by a non-circular cross section of MscL. In comparison to Fig. 5(A), the gating curves in Fig. 5(B) are shifted to a larger tension into the regime of the measured gating tension 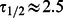 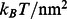 [30], [33] for which 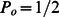 in Eq. (1). Moreover, for the parameter values used in Fig. 5(B), the gating tension associated with the structural models in Fig. 1 and Refs. [39], [44] is generally larger than the gating tension of the cylinder model of MscL. Contrary to Fig. 5(A), Fig. 5(B) implies that the hexameric clover-leaf model of MscL gates at a smaller tension than the pentameric clover-leaf model of MscL. Similarly as Fig. 5(A), however, Fig. 5(B) predicts that the tetragonal model of MscL gates at a larger membrane tension than the corresponding pentagonal model. Moreover, Figs. 5(A) and 5(B) both imply that for a pentagonal shape of MscL in the closed state, and a pentameric clover-leaf shape in the open state, the gating tension is increased relative to most other scenarios suggested by Fig. 1 and Refs. [39], [44], with the reverse result for the case of a closed pentameric clover-leaf shape and an open pentagonal shape of MscL. Collectively, Fig. 5 shows that, even if only membrane contributions to the gating energy are considered, different oligomeric states and hydrophobic shapes of MscL yield considerable and distinctive modifications of the gating characteristics of MscL.

### Gating curves: Systematic trends

In analogy to Fig. 4, we have also carried out a systematic comparison between the gating characteristics associated with different oligomeric states of MscL for the polygonal and clover-leaf boundary curves inspired by Fig. 1 and Refs. [39], [44] (see Fig. S4). For this comparison we used, as in Fig. 5A, the same hydrophobic mismatch for closed and open states of MscL [51], [52]. As already suggested by the results in Fig. 4 we found that, for clover-leaf shapes, higher-order oligomeric states gate at a smaller membrane tension. Moreover, depending on the oligomeric state considered, clover-leaf membrane inclusions can gate at a smaller or at a larger tension than the cylinder model of MscL. For polygonal shapes, higher-order oligomeric states are also found to gate at a smaller membrane tension than lower-order oligomeric states but, in contrast to clover-leaf shapes, polygonal channels always gate at a larger tension than the cylindrical reference shape. These features of the gating characteristics of polygonal membrane inclusions do not change if inclusions of equal circumference, rather than equal cross-sectional area, are compared, although the differences in the gating tensions associated with the various oligomeric states of polygonal inclusions become less pronounced.

## Discussion

Inspired by structural studies of MscL [24]–[27], [33], [35]–[47] we have determined the membrane deformation energy associated with a variety of oligomeric states and hydrophobic shapes of MscL. Our analysis focused on the limit of weak perturbations about the cylinder model of membrane proteins, which was employed previously to study bilayer-protein interactions for MscL [51]–[54] as well as for a number of other membrane proteins [11]–[15]. It would desirable to complement the analytic approach developed here with numerical schemes allowing the accurate solution of the elastic membrane equations for complicated protein shapes. Such numerical schemes will be crucial for connecting membrane-mechanical models of bilayer-protein interactions more closely to the shapes of real membrane proteins. Moreover, in our analysis we have focused solely [11]–[15], [51]–[54] on contributions to the total gating energy due to thickness deformations of the bilayer membrane. In particular, we did not consider contributions to the free energy difference between the open and closed states of MscL due to changes in the internal protein free energy. While it has been argued [51]–[54] that, at least for some strains of MscL [27], the thickness deformation energy may play a dominant role in MscL gating, other contributions to the free energy budget must generally be considered.

Our mathematical approach for determining the energetic cost of membrane deformations associated with different oligomeric states and hydrophobic shapes of MscL is general and directly applicable to other membrane proteins. Thus, the methodology developed here establishes a quantitative relationship between the oligomeric state and hydrophobic shape of a membrane protein and the elastic energy required to accommodate the membrane protein within the lipid bilayer membrane. However, the quantitative details of our predictions depend on the parameter values characterizing the hydrophobic shape of the membrane protein under consideration. In particular, crucial inputs for our model are the hydrophobic thickness and cross section of membrane proteins. Recent experimental results [3]–[6], [9], [10] on bilayer-protein interactions suggest that it may be feasible to substantially refine these model inputs to arrive at a more realistic description of protein-induced membrane deformations. For instance, we assumed here that the hydrophobic surface of MscL is perpendicular to the bilayer membrane and of a constant thickness, while a more realistic description of bilayer-MscL interactions would allow [64] for variations in the hydrophobic thickness of MscL along the bilayer-MscL interface.

The physiologically relevant oligomeric states and molecular structures of MscL remain a matter of debate [26], [35]–[37], with tetrameric [46], pentameric [40], and hexameric [39] states of MscL having been reported. The oligomeric state and molecular structure of MscL have so far mainly been studied [24]–[27], [33], [35]–[47] using crystallographic, biochemical, and computational approaches. Our results suggest that, for cases in which there is a significant membrane contribution to the gating energy, functional properties of MscL, such as the predicted discrepancies in the gating energy and gating tension between different oligomeric states and structural models of MscL [24]–[27], [33], [35]–[47], may also be used to shed light on the physiologically relevant oligomeric states and molecular structures of MscL. While we have illustrated our approach for MscL, the methods developed here are general and applicable to other membrane proteins. We predict that the oligomeric state and hydrophobic shape of a membrane protein are reflected in the energetic cost of the lipid bilayer deformations necessary to accommodate the protein within the membrane. Thus, our results suggest that, in addition to the hydrophobic mismatch between membrane proteins and the surrounding lipid bilayer [11]–[17], the symmetry and shape of the hydrophobic cross section of membrane proteins, and resulting structure of elastic membrane deformations, play an important role in the regulation of protein function by bilayer membranes.

## Models and Methods

### Elastic model of mechanosensitive gating

In accordance with the standard framework for describing elastic bilayer-protein interactions [11]–[15], [51]–[63], we model MscL as a rigid membrane inclusion inducing bilayer deformations as a result of a hydrophobic mismatch between lipid bilayer and membrane protein. In mathematical terms, the lipid bilayer is represented within the Monge representation of curved surfaces using the functions 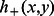 and 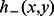, which define the positions of the hydrophilic-hydrophobic interface at the Cartesian coordinates 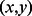 in the top and bottom (outer and inner) membrane leaflets. Focusing on thickness deformations induced by MscL [14], [51]–[54], we consider the elastic energy [18], [20], [56]

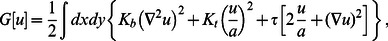
(4)where the thickness deformation field 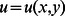 is defined by
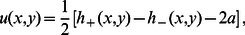
(5)in which 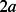 is the equilibrium thickness of the unperturbed bilayer, 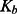 is the bending rigidity, 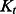 is the stiffness associated with thickness deformations, and 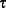 is the membrane tension. Energy functionals of the form in Eq. (4) have been employed in a range of studies [11]–[15], [51]–[63] of membrane deformations induced by MscL as well as other membrane proteins.

The terms 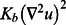 and 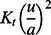 in Eq. (4) provide lowest-order descriptions of the energetic cost of membrane bending and compression or expansion of the lipid bilayer, respectively. For generality we allow for the two tension terms 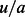 and 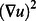 in Eq. (4), which were employed previously to describe the effects of membrane tension on lipid surface area [18], [53], [54] and on membrane undulations [18]–[20], [51], [52], [56]. While Eq. (4) provides a simple description of protein-induced membrane deformations, more sophisticated models of membrane deformations can be developed [20], [52], [55]–[59] in order to account for detailed properties of lipid bilayers such as lipid structure and spontaneous curvature. Finally, the elastic model of bilayer membranes in Eq. (4) is completed by accounting for the midplane deformations
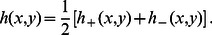
(6)To leading order, midplane deformations decouple from thickness deformations in the total membrane elastic energy [56]. It was found previously [14], [51]–[54] that energetic contributions to MscL gating due to midplane deformations can generally be neglected relative to energetic contributions due to thickness deformations, and we therefore focus here on Eq. (4).

The specific properties of MscL enter Eq. (4) through the boundary conditions at the bilayer-MscL interface [12]–[15], [51]–[54]. For convenience, we specify these boundary conditions along some boundary curve 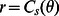 using polar coordinates:
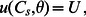
(7)

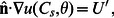
(8)where 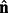 is the unit normal vector along the bilayer-inclusion interface. If MscL is described as a cylindrical membrane inclusion [51]–[54], 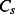 is a constant and 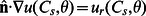. The quantity 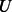 corresponds to one-half the hydrophobic mismatch between MscL and the surrounding lipid bilayer, and 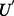 corresponds to the gradient of the thickness deformation field at the bilayer-inclusion interface. We denote the values of 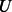 and 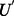 associated with the closed and open channel states by 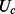 and 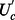, and by 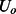 and 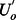, respectively. The crystallographic structure of the closed state of MscL suggests [40], [41], [54] 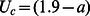 nm, while it has been proposed [41], [47], [54] that 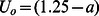 nm for the open state of MscL. To our knowledge, no experimental estimates of the values of 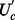 and 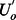 are available for MscL but, within the membrane-mechanical model of MscL gating, these parameters were found previously [51]–[54] to play a minor role compared to 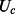 and 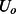, and are commonly set to zero. We set 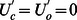 in all calculations presented here. An approach alternative to that in Eq. (8) would allow [55], [57]–[63] for a free contact slope along the bilayer-inclusion interface.

The membrane-mechanical model of bilayer-MscL interactions outlined above yields a qualitative framework for understanding MscL gating, is in broad agreement [14], [51]–[54] with available experimental data, and provides a machinery for making quantitative predictions. In particular, within the framework of this model, MscL gating is understood on a qualitative level as driven by two competing physical mechanisms. On the one hand, closed channels generally leave a smaller elastic deformation footprint in the membrane, which makes the closed state favorable compared to the open state. On the other hand, in membranes under tension, the increase in membrane area associated with open channels makes this state favorable compared to the closed state. Put differently, MscL gating harnesses the mechanical properties of lipid bilayers for channel function, which penalize the more pronounced membrane deformations which are generally necessary to accommodate larger channels, but favor the relaxation of the tension-inducing loading device [26], [54] brought about by an increased channel area. This physical picture of mechanosensitive gating [14], [51]–[54] relies on the implicit assumption that, in the closed state of MscL, 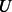 and 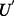 in Eqs. (7) and (8) are of a similar or smaller magnitude as in the open state of MscL.

While the elastic model in Eq. (4) provides a general description of membrane shape [12]–[15], [18]–[20], quantitative tests of the relevance of this model for mechanosensitive gating rely [14], [51]–[54] on comparing theoretical estimates of 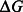 to measured values of 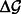. In the absence of reliable measurements of 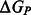 in Eq. (2), and presence of large experimental uncertainties, any such comparison can only be of broad character. In the simplest case, the closed and open states of MscL are assumed to take cylindrical shapes with the same hydrophobic thickness, which is then fitted to experimental data. In agreement with the experimental results in Ref. [27], it is thus found [51], [52] that 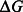 varies from 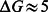 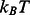 to 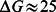 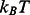 as the lipid tail length is varied from 16 carboxyl groups to 20 carboxyl groups, and that this variation approximately takes the shape of a quadratic function. This result is obtained at zero tension with the fitted hydrophobic mismatch 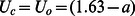 nm, which corresponds to a hydrophobic thickness of MscL matching a PC12 bilayer and lies in between the aforementioned values of 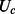 and 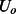 proposed on the basis of the crystallographic structure of the closed state of MscL [40], [41] and molecular modeling of the open state of MscL embedded in doped bilayers [41], [47]. For a finite tension 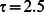 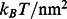, which approximately corresponds to the critical gating tension at which 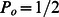 in Eq. (1), one finds [54] for the cylinder model of MscL with the values of 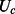 and 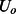 proposed on the basis of structural studies of MscL [40], [41], [47] that 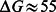 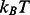 for a model lipid bilayer. This estimate does not involve any free parameters, and agrees quite well with the corresponding experimental estimate 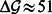 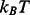 in Refs. [30], [33]. We employ the fitted value 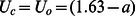 nm [51], [52] in Figs. 2–4 and 5(A), as well as Figs. S2, S3, S4, for our systematic study of the effect of protein shape on the membrane deformation energy and gating tension. This parameterization of bilayer-MscL interactions allows us to avoid any spurious effects resulting from different hydrophobic mismatches in the closed and open channel states. In Fig. 5(B) we use the estimates 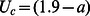 nm and 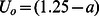 nm suggested in Refs. [40], [41], [47], [54].

### General solution of the elastic model

We follow Refs. [11]–[15], [51]–[63] and use Eq. (4) with the boundary conditions in Eqs. (7) and (8) as our basic model of the membrane deformations induced by MscL. The Euler-Lagrange equation associated with Eq. (4) is given by
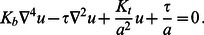
(9)To proceed, we introduce the function
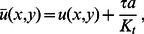
(10)in terms of which Eq. (9) reduces to
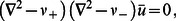
(11)where
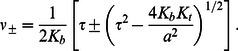
(12)The solution of Eq. (11) is of the form [11], [65]

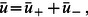
(13)where 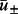 are solutions of the Helmholtz equations
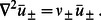
(14)For the exterior of a circle of radius 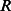, the above Helmholtz equations are readily solved by separation of variables [65], [66]. Thus, for the exterior of a circle, the solution of Eq. (11) can be written as the Fourier-Bessel series
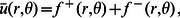
(15)in which
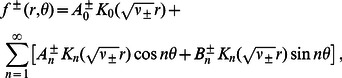
(16)where 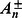 and 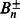 are constants, 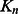 are modified Bessel functions of the second kind, and we have assumed that membrane deformations decay away from the membrane inclusion [57]. At each order in the Fourier-Bessel series in Eq. (15), two boundary conditions at the membrane-inclusion interface are required to fix all constants 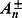 and 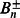.

Boundary curves are obtained by fitting the Fourier representation of 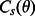,
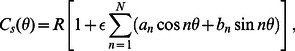
(17)in which we take
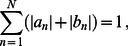
(18)and 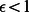, to the transmembrane cross sections of MscL in Fig. 1 and Refs. [39], [44]. We focus here on the weak perturbation limit of Eq. (17) and only consider leading-order terms in 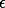.

The molecular structures in Fig. 1 and Refs. [39], [44] suggest two basic families of 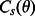 as models of the hydrophobic cross section of MscL: polygonal boundary shapes and clover-leaf boundary shapes. Polygonal shapes are obtained using the Fourier representation of regular 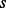-gons in the complex plane [67],
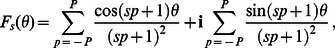
(19)in which 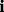 is the imaginary unit and the tetragonal and pentagonal oligomeric states in Fig. 1(A) and Ref. [44] correspond to 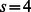 and 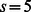, respectively. Higher orders of 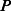 in Eq. (19) yield increasingly sharp polygonal corners. For all polygonal shapes in this manuscript we considered terms up to 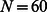 in Eq. (17). As described in the Results section, all parameters in Eq. (17) are then fixed for polygonal shapes by setting the areas of polygonal shapes equal to the cross-sectional areas of closed and open MscL suggested by structural studies [27], [33], [40]–[45], [47] and used in previous membrane-mechanical models of MscL gating [51], [52], [54].

The clover-leaf shapes in Fig. 1 are obtained using boundary curves of the form
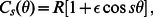
(20)where the pentameric and hexameric clover-leaf shapes in Fig. 1(B) and Ref. [39] correspond to 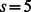 and 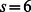, respectively. As for polygonal shapes, the overall coefficient 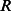 in Eq. (20) is determined by fixing the area of clover-leaf shapes in closed and open channel states [27], [33], [40]–[45], [47], [51], [52], [54]. For the clover-leaf shapes considered in Figs. 2–5, we determined 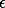 through fits to the models of MscL shape shown in Fig. 1 and Ref. [39], yielding 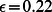 (closed pentameric clover-leaf shape), 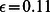 (open pentameric clover-leaf shape), and 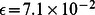 (closed and open hexameric clover-leaf shapes). For the model clover-leaf shapes shown in Figs. S1, S2, S3, S4 we used 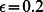 for closed states and 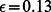 for open states so that the amplitude of perturbations about the cylindrical reference shape, 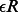, took the same magnitude in closed and open states.

In general, 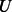 and 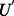 in the boundary conditions in Eqs. (7) and (8) at 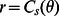 may both exhibit an angular dependence, and our approach is able to handle such cases. Here we focus on the effect of deviations from the circular shape on the elastic membrane deformations induced by MscL. For simplicity, we therefore take 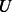 and 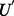 to be constants. Assuming small deviations 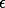 from circularity in Eq. (17), we use a perturbative approach and expand [68] 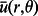 at the boundary curve 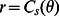 around 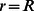 to leading order in 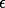,
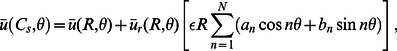
(21)in which
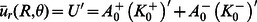
(22)from the general solution in Eq. (15) to 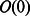 in 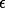, where
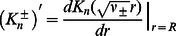
(23)for 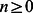. Note, in particular, that any term in Eq. (15) involving an angular dependence must at least be of 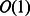 in 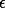. Similarly,
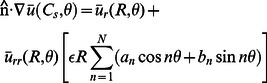
(24)to leading order in 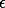, in which
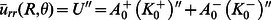
(25)from the general solution in Eq. (15) to 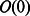 in 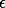, where
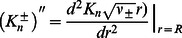
(26)for 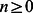, and 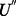 is determined by the 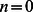 terms in Eq. (15).

Thus, using Eqs. (21) and (24), we can recast the boundary conditions in Eqs. (7) and (8) for non-cylindrical inclusions as boundary conditions for cylindrical inclusions of variable hydrophobic thickness,
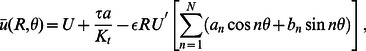
(27)

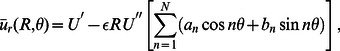
(28)to leading order in 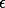. Matching Eqs. (27) and (28) with Eq. (15) at each order in the Fourier-Bessel series, we find
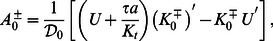
(29)

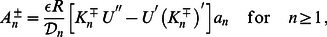
(30)

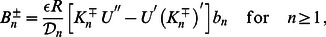
(31)where, for 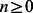, 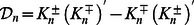 and 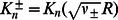. Equations (29)–(31) together with Eq. (15) constitute, in the limit of weak perturbations about cylindrical inclusion shapes, the general solution of the membrane deformation profile for arbitrary oligomeric states of MscL.

The membrane deformation energy associated with the equilibrium deformation profile in Eq. (15) with Eqs. (29)–(31) is obtained by evaluating the surface integral in Eq. (4). To this end, we note from Eq. (11) that
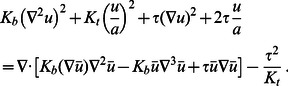
(32)Hence, we can use Gauss's theorem in the plane to transform the surface integral in Eq. (4) to a line integral:
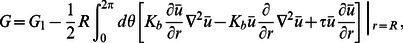
(33)where 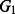 is a constant. For simplicity, we choose the zero of the energy such that 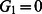.

To evaluate the integrals in Eq. (33) it is convenient to note that 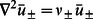. Substituting the Fourier-Bessel series in Eq. (15) into Eq. (33) then generates integrals of the form
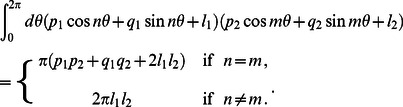
(34)Thus, we find the elastic thickness deformation energy
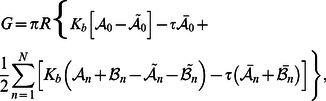
(35)where
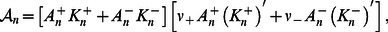
(36)

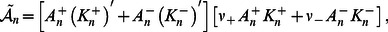
(37)

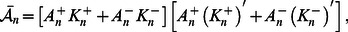
(38)

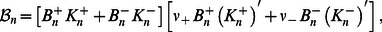
(39)

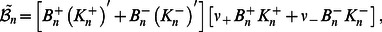
(40)

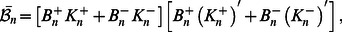
(41)for 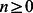. Equation (35) with Eqs. (36)–(41) and Eqs. (29)–(31) provides the general solution of the thickness deformation energy in Eq. (4) for arbitrary oligomeric states of MscL in the limit of weak perturbations about cylindrical inclusion shapes.

The deformation profiles in Fig. 2 were obtained from Eq. (15) with Eqs. (29)–(31), the energy curves in Figs. 3, 4, S2, and S3 were obtained from Eq. (35) with Eqs. (36)–(41) and Eqs. (29)–(31), and the gating curves in Figs. 5 and S4 were obtained from Eq. (1) together with Eq. (35), Eqs. (36)–(41), and Eqs. (29)–(31). For all plots we used the elastic moduli [54] 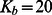 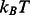 and 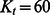 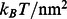, with 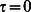 for Figs. 2–4, S2, and S3. The results in Figs. 2–4, 5(A), and S2, S3, S4 were obtained with 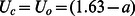
nm [51], [52]. For Fig. 5(B) we used the estimates 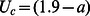
nm and 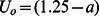
nm [40], [41], [47], [54]. We used a bilayer hydrophobic thickness corresponding to PC14 lipids for Fig. 1, to PC18 lipids for Figs. 4, 5(A), and S4, and to PC14 lipids for Fig. 5(B). We related membrane hydrophobic thickness to PC lipid tail length using the simple interpolation described in Ref. [51].

### Accession numbers

The primary accession numbers (in parentheses) from the Protein Data Bank are: Pentameric MscL (2OAR, formerly 1MSL; Resolution of 3.50 Å; Ref. [40]) and tetrameric MscL (3HZQ; Resolution of 3.82 Å; Ref. [46]).

## Supporting Information

Figure S1
**Cross sections of model inclusion shapes.** Boundary curves 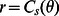 in Eq. (17) which (A) deviate from a circle by a single term 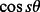 and (B) approximate regular polygons. Our point of reference for the inclusion shapes is a cylinder of radius 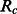 with 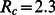 nm, which previous calculations [14], [51], [52], [54] employed as a model of the closed state of MscL. The inclusion shapes shown are inspired by the structural models of MscL in Fig. 1 and Refs. [39], [44] of the main text. The solid curves in panels (A) and (B) denote membrane inclusions with cross-sectional area 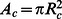, while the dashed curves in panel (B) denote polygonal shapes with circumference 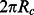.(EPS)

Figure S2
**Membrane deformation energy of model inclusion shapes.** Thickness deformation energy in Eq. (35) induced by the inclusion shapes in Fig. S1 as a function of lipid tail length for (A) clover-leaf boundary curves and (B) polygonal boundary curves. The shaded region in panel (A) denotes the membrane deformation energy associated with the cylinder model of MscL for the range of radii indicated in the insets and in Fig. S1(A). The solid curves in panels (A) and (B) correspond to polygonal shapes with cross-sectional area 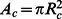, while the dashed curves in panel (B) correspond to polygonal shapes with circumference 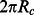. We used identical values of the hydrophobic inclusion thickness for all model shapes shown.(EPS)

Figure S3 **Gating energy of model inclusion shapes.** Difference in thickness deformation energy between the open and closed states of generalized shapes of MscL obtained from Eq. (35) for the boundary shapes shown in Fig. S1. We use the same parameter values and labeling conventions as in Fig. 3(B) of the main text.
(EPS)

Figure S4 **Gating probability of model inclusion shapes.** Membrane contribution to the opening probability of generalized shapes of MscL obtained from Eq. (1) together with Eq. (35) for the boundary shapes shown in Fig. S1. We use the same parameter values and labeling conventions as in Fig. 5(A) of the main text.
(EPS)
